# Heart Failure Emergency Readmission Prediction Using Stacking Machine Learning Model

**DOI:** 10.3390/diagnostics13111948

**Published:** 2023-06-02

**Authors:** Md. Sohanur Rahman, Hasib Ryan Rahman, Johayra Prithula, Muhammad E. H. Chowdhury, Mosabber Uddin Ahmed, Jaya Kumar, M. Murugappan, Muhammad Salman Khan

**Affiliations:** 1 Department of Electrical and Electronics Engineering, University of Dhaka, Dhaka 1000, Bangladesh; 2 Department of Electrical Engineering, Qatar University, Doha 2713, Qatar; 3 Department of Physiology, Faculty of Medicine, University Kebangsaan Malaysia, Kuala Lumpur 56000, Malaysia; 4 Intelligent Signal Processing (ISP) Research Lab, Department of Electronics and Communication Engineering, Kuwait College of Science and Technology, Block 4, Doha 13133, Kuwait

**Keywords:** heart failure, emergency readmission, machine learning, electronic health data, stacking classification

## Abstract

Heart failure is a devastating disease that has high mortality rates and a negative impact on quality of life. Heart failure patients often experience emergency readmission after an initial episode, often due to inadequate management. A timely diagnosis and treatment of underlying issues can significantly reduce the risk of emergency readmissions. The purpose of this project was to predict emergency readmissions of discharged heart failure patients using classical machine learning (ML) models based on Electronic Health Record (EHR) data. The dataset used for this study consisted of 166 clinical biomarkers from 2008 patient records. Three feature selection techniques were studied along with 13 classical ML models using five-fold cross-validation. A stacking ML model was trained using the predictions of the three best-performing models for final classification. The stacking ML model provided an accuracy, precision, recall, specificity, F1-score, and area under the curve (AUC) of 89.41%, 90.10%, 89.41%, 87.83%, 89.28%, and 0.881, respectively. This indicates the effectiveness of the proposed model in predicting emergency readmissions. The healthcare providers can intervene pro-actively to reduce emergency hospital readmission risk and improve patient outcomes and decrease healthcare costs using the proposed model.

## 1. Introduction

A significant proportion of hospitalizations and deaths among elderly individuals is associated with heart failure (HF). A physiological condition of HF is either not enough blood flow from the heart to meet the body’s metabolic demands or a compensatory neurohormonal response resulting in increased pressure in the left ventricle [1]. HF has been further divided into three subtypes based on ejection fraction, natriuretic peptide levels, structural heart disease, and diastolic dysfunction in recent years. The three subtypes of heart failure are heart failure with a reduced ejection fraction (HFrEF), heart failure with a preserved ejection fraction (HFpEF), and heart failure with a mid-range ejection fraction (HFmrEF) [2].

### 1.1. Background

In the United States, more than a million patients are hospitalized each year because of HF. Patients with HF often require hospital readmissions, which cost on average USD 23,000 per hospitalization. There is an estimated increase of USD 70 billion in costs for these admissions by 2030 [3,4,5,6]. The number of people suffering from heart failure in the US is currently over 6.2 million [7]. The prevalence of this disease is predicted to increase by 46% by 2030, reaching over 8 million [8]. Despite advances in medical treatment, hospital readmission rates remain high, with over half of patients readmitted within six months of discharge [9,10,11]. It is imperative to understand why some hospitals perform well while others struggle in order to deal with this issue. Previous studies have examined the first readmission, but not all the patients experience re-hospitalizations after the initial hospitalization for HF. Several interventions for patients with HF have been shown to reduce the risk of readmission by providing higher-quality care [12,13].

Predicting hospital readmission of heart failure patients is crucial for several reasons, including improving the quality of care, reducing healthcare costs, optimizing resource allocation, and enhancing patient outcomes [14]. High rates of readmission may indicate inadequate care during the initial hospitalization and discharge planning, leading to negative impacts on patient health and well-being. Predictive models can help healthcare providers identify patients at the highest risk of readmission and allocate resources effectively to improve outcomes. Additionally, predicting readmission risk can help healthcare organizations meet performance metrics and identify areas for improvement in care delivery. It has been observed that the elderly population with a previous HF diagnosis and a history of cardiac revascularization have a higher chance of unplanned readmission [15].

### 1.2. Related Works

A review by Ouwerker et al. of 117 models found that blood urea nitrogen and sodium were the strongest predictors for HF hospitalization [16]. Four subgroups of models were identified, with prospective registry-type studies using a large number of clinical predictor variables being the most accurate in predicting mortality. Blood sugar levels are also found to be crucial for HF patients [17]. The Center for Medicare and Medicaid Services (CMS) has emphasized reducing readmission rates to reduce costs and improve care quality [18]. When examining claims data from 2003–2004, it was discovered that a significant number of Medicare beneficiaries were readmitted to the hospital within 30 days (19.6%) and 90 days (34.0%) after being discharged. Both medical and surgical patients were affected, but medical patients had a higher readmission rate, accounting for 77.1% of the rehospitalizations. The top conditions associated with high 30-day readmission rates were heart failure (26.9%), psychoses (24.6%), recent vascular surgery (23.9%), chronic obstructive pulmonary disease (22.6%), and pneumonia (20.1%) [19]. Despite efforts to reduce readmissions, risk-adjusted 30-day readmission rates among Medicare beneficiaries have remained fairly stable over the past decade. According to a study conducted by Artetxe et al., it has been found that machine learning methods can enhance the accuracy of predictions as compared to conventional statistical techniques [20]. The researchers noted that logistic regression (LR) and survivability analysis are the frequently utilized techniques for developing models to anticipate readmission rates. A machine learning approach to HF treatment, including diagnosis, classification, readmissions, and medication, has the potential to improve treatment quality and cut costs [21].

Data extracted from routinely collected electronic healthcare records (EHRs) have been used in a few studies. Based on clinical and physiological data available in EHR systems, Beecy et al. developed a comprehensive approach to predicting 30-day unplanned readmissions and all-cause mortality (ACM) [22]. They designed three different predictive models, using an extreme gradient boosting (XGBoost) classifier, which were based on index admission, index discharge, and feature-aggregation. Due to the lack of previous data on heart failure rates in China, Zhang Z. et al. studied the symptoms of HF patients admitted to Zigong Fourth People’s Hospital between December 2016 and June 2019. Based on their work, baseline clinical characteristics (including body temperature, pulse, respiration rate, body mass index (BMI), etc.) and primary drug categories were recorded on admission. The majority of other studies focus only on 30-day emergency readmission rates, which may not represent the majority of patients. This study used follow-up periods of 28 days, 3 months, and 6 months for better statistical analysis and interpretation [23]. Data mining techniques and significant features have been found to boost the accuracy of cardiovascular survivor prediction by Ishaq et al. By using synthetic minority oversampling technique (SMOTE) to offset the imbalance in the dataset, they achieved a remarkable accuracy of 0.926 using the ExtraTree (ET) classifier algorithm [24]. A deep-learning-based approach was performed by Golas et al., as they developed a risk prediction model for hospital readmission including structured and un-structured data, constructed using deep unified networks (DUNs) to avoid over-fitting [25]. The DUNs model had the best performance after 10-fold cross-validation, reaching an AUC of 0.705  ±  0.015 and 76.4% accuracy. Another deep-learning-based study by Liu et al. proposed a CNN model with an F1 score of 0.756 and 0.733 in general readmission prediction and 30-day readmission prediction, respectively [26].

### 1.3. Research Objectives and Contribution

Our aim was to determine whether patients with heart failure who had previously undergone treatment and been discharged would be likely to return to the hospital in the event of an emergency. In order to accomplish this goal, an EHR dataset of HF patients was used. The dataset included comprehensive information about the patient’s physical and medical conditions, including symptoms and signs of HF, and their discharge status. We used 13 variants of classical machine learning (ML) models and a stacking machine learning (or meta-learner)-based model. In this study, the following novel contributions are made:

- This study examines a large dataset to predict emergency readmissions for heart failure patients.
- Thirteen variants of classical machine learning models were examined using different feature selection techniques along with a stacking machine learning model.
- Based on nine biomarkers, this study produced very good predictions for emergency department readmission for heart failure patients.
- LACE-related feature incorporation to the feature set provided a significant performance boost in the study to predict hospital readmission due to heart failure emergency.

The rest of the paper is organized as follows: Section 2 discusses the methodology of the study by describing the datasets used in this paper and the details of data pre-processing stages for machine learning classifiers and the stacking machine learning algorithm. Section 3 discusses the result of the classification models and the stacking machine learning model. Finally, the article is concluded in Section 4.

## 2. Methodology

**Figure 1:**
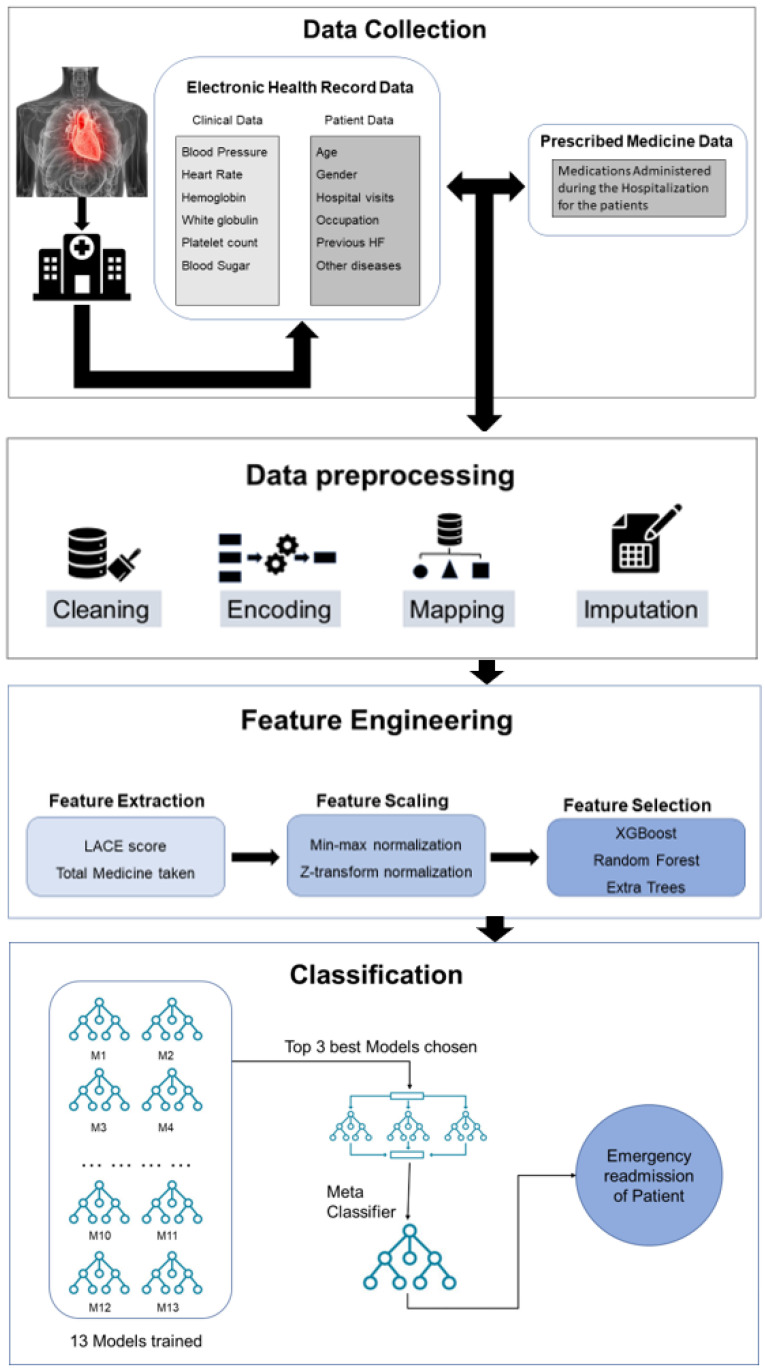
General overview of the methodology.

This study attempts to predict the emergency readmission of HF patients after discharge from a medical facility using a dataset that is made publicly available in Physionet [27]. The dataset has three different forms of data: the electronic health record, medication, and dictionary. An extensive analysis of the data led to the selection of the most important features for the model. Data from electronic health records and drug databases were combined, and feature engineering was performed. An important parameter, a LACE score, was added to the feature list for identifying readmissions cases reliably. Thirteen variants of classical ML models were trained with a reduced feature set selected by a feature-ranking technique. A stacking ML model was trained using the top three classical ML models, which improved the overall performance. A solid and reliable model for forecasting HF patients’ emergency readmissions was developed as a result of the approach. The flowchart in Figure 1 provides a general picture of how the study was handled.

### 2.1. Dataset Description

We collected this EHR dataset from a public source available from Physionet [27]. The EHR dataset was collected by Zhang Z. et al. to study the symptoms of HF patients admitted to Zigong Fourth People’s Hospital between December 2016 and June 2019. The study was approved by the ethics committee of Zigong Fourth People’s Hospital (Approval Number: 2020-010). As there was a scarcity of datasets available on HF, the researchers targeted Zigong Fourth People’s Hospital in Sichuan, China. They extracted data from a routinely collected EHR. In addition, follow-up data were collected on hospital admissions, mortality rates, and required follow-up visits at 28 days, 3 months, and 6 months after discharge. In the event that a patient had a medical emergency and was unable to travel to the clinical facility, their information was collected by phone. Participants’ data were collected between December 2016 and June 2019, providing researchers with an in-depth look at their health conditions and outcomes over time. Researchers were able to create more effective medications and interventions after understanding HF and the variables that affect patient outcomes.

The clinical data were collected on the day of the subject patients’ hospital admission. Clinical data included information from a variety of medical tests, including body temperature, pulse, respiration rate, systolic and diastolic blood pressure, mean arterial blood pressure, weight, height, body mass index (BMI), type of HF, New York Heart Association (NYHA) cardiac function, and Killip Grade (Class 1: no rales, no third heart sound; Class 2: rales in 0.5 lung fields or the presence of a third heart sound). This sub-dataset contained 2008 records and 166 features excluding a redundant index column. Information related to admission, discharge, and medical test reports was included in these features.

A separate dataset included information on the medications consumed by the subject individuals while in the hospital. There was no mention of the time that the drugs were taken; only the medications were mentioned. In the dataset, there were multiple instances of the same medicine because values were inputted every time a medication was taken, indicating more than one dose was administered. This sub-dataset contains 15,362 records and 2 feature columns, including inpatient number and medication name. We were able to combine the two datasets into one by combining the inpatient number in each of the sub-datasets.

### 2.2. Statistical Analysis

The variables of this study were statistically analyzed using Python 3.8. Ages were classified according to decades. There were eight age categories from 19 to 110. Three categories were available for the readmission period: 28 days, three months, and six months. By applying Pearson correlation coefficient (PCC) for each feature with other features, we were able to exclude highly correlated features with a score greater than 0.80 that were highly similar to the label data and could lead to overfitting our model. PCC formula is given as below [28]:

(1) $$r=\frac{\sum_{i=1}^{n}\left(x_{i}-\bar{x}\right)\left(y_{i}-\bar{y}\right)}{\sqrt{\sum_{i=1}^{n}{\left(x_{i}-\bar{x}\right)}^{2}}\sqrt{\sum_{i=1}^{n}{\left(y_{i}-\bar{y}\right)}^{2}}}$$

where r, $x_{i}$, $\bar{x}$, $y_{i}$, $\bar{y}$ indicate the Pearson correlation coefficient, feature value, mean feature value, other feature value, other mean feature value. Here, features x and y have a length of ***n***.

Table 1 shows the characteristics of the patients’ return to the Emergency Department within six months, the NYHA cardiac function classification, the use of Digoxin tablets, the presence of a magnesium serum, and the presence of potassium ions. The 1951 patients had a return home rate of 67.5% at 1318, a hospitalization rate of 22.5% at 436, an unknown rate of 9.8% at 192, and a mortality rate of 0.2% at 5. Due to the small number of patients who passed away in hospitals, they were excluded from the dataset to avoid low-quality features. The number of male patients was 845 (42.09%) and the number of female patients was 1163 (57.91%). Approximately 7.657 medications were consumed by each patient, on average, with a standard deviation of 2.4204. Among 2008 patients, 92.8% (1865) had myocardial infarction and 93.2% (1872) had congestive HF. The most common disease was connective tissue disease (99.7%).

### 2.3. Preprocessing

A physical examination was conducted on each patient to verify the accuracy and authenticity of the dataset. This included height, weight, body mass index (BMI), type of heart failure, and heart condition. However, certain anomalies were discovered when the data were examined. The height, weight, and BMI measurements of some patients were atypical, and some of these measurements were recorded as 0. The anomalous values were not consistent with the known range of human measurements. As an example, the shortest individual ever listed in the Guinness Book of Records, who was about 0.54 m tall, did not fit with certain height numbers that were especially unusual, such as 0.35 m [29]. The ages of the patients were reviewed to confirm the accuracy of these data. Analysis revealed that 0.697% of discharged patients died in the hospital after they were discharged. These records were excluded from the dataset.

Height and weight outliers were missing data that were filled in with 0 and NaNs (Not a Number) were substituted with 0. Additionally, the ages of the patients were listed in decade form instead of as exact numbers. As a result, the age range was converted from an object type to an integer by replacing it with the average of the range. A total of 15 columns with NaN values exceeding 65% were removed from the dataset in order to avoid overfitting and skewed results. We removed columns for blood temperature, blood gas, and leukemia because they would not be relevant to the model’s training and would interfere with standardizing and normalizing the dataset.

The objective of this project was to develop a model for discharged patients to be readmitted in the event of an emergency. Therefore, we needed to select the right predicting label file. A total of two columns matched the target. In one column, patients were listed who got readmitted after days of prior admission; in the other, the number of days after the initial admission that the patients had to be in the emergency department within 6 months was listed. The correlation between these two columns was calculated using Equation (1), and we found that it was 0.99. Therefore, any patient who needed to be admitted again would have to go through the emergency department. In this regard, the ‘re.admission.time.days.from.admission.’ column is set as the label file. All values greater than 0 in that column were transformed into 1, indicating the patient had been readmitted, and NaN values were transformed into 0, meaning the patient had not been readmitted. We dropped the ‘time.to.emergency.department.within.6.months’ variable to prevent overfitting.

#### 2.3.1. Encoding

Despite having a plethora of categorical columns, the EHR dataset would be difficult to use in its current form without encoding. An encoding process was carried out on the entire dataset in order to prepare it in a way that can be easily fed into the classification models in order to have greater accuracy. Each distinct categorical item was represented by an ordinal number. Drug datasets used an additional encoding method, where a new vector was integrated for each distinct categorical value. It is now possible to concatenate the EHR datasets. The medication dataset’s form was altered due to encoding, which also created 25 new columns with the parent ID column.

#### 2.3.2. Dataset Mapping

After encoding the dataset in two different encoding methods, it was necessary to merge the datasets into a single dataset, which was achieved through dataset mapping. Each EHR dataset and medication dataset had the same Patient ID to identify each patient individually. There were multiple entries under the same Patient ID in the medication dataset. Each patient taking the same medicine multiple times was summed under a single patient in a column, so the recurrence of Patient ID was eliminated. The data had been merged between two datasets based on Patient ID. Therefore, all drug name columns were concatenated with the EHR dataset, and the shape of the dataset was the same as that of the EHR dataset.

#### 2.3.3. Missing Data Imputation

Due to the practical difficulty of collecting all information from a subject, missing data were likely in the dataset. The missing values were represented by NaN. Due to the fact that machine learning models are mathematical in nature, they are not capable of processing data of any other kind. It is therefore imperative to adopt essential safeguards. The removal of NaN values is one way to deal with the missing values, but this approach would result in substantial data loss and rather poor ML performance. Alternatively, NaN values can be replaced with appropriate values based on the records’ pattern. By using the entire feature data, the data imputation approach fills in the missing values [30]. The inclusion of missing indicator variables is preferred in some studies [31,32], while it is also controversial [33] in medical research. Several other studies totally excluded missing data in order to prevent high bias [34,35,36].

In our study, we carefully examined the EHR dataset and found that many key predictors had missing parameters. In order to prevent data loss, an important data imputation method was used. In this study, MICE, or Multivariate Imputation by Chained Equations, was employed as the imputation technique [37,38]. As part of this method, both mean and regression methods are applied successively within a feature column to make the missing records comparable to other records that have similar values. Using this imputation method, the analyzed data were clinical data, which made the imputation method successful.

#### 2.3.4. LACE Feature Extraction

The LACE index, also known as Length of stay, Acuity of admission, Comorbidities, and Emergency department use, is widely used in the medical field in the US and Canada [39,40,41]. This mathematical model was developed by van Walraven et al. [42] to predict the risk of unplanned readmission or death within 30 days after discharge from the hospital for medical and surgical patients. Four main factors can help healthcare providers make informed decisions about patient care [43]. They can identify patients who may require extra support after discharge, prevent unplanned readmissions, and improve patient outcomes.

After connecting the one-hot encoded data with the EHR information, we identified the medications taken by the subject patients. Because HF groups are usually associated with a high number of prescription drugs, we introduced the total number of medications patients take to represent their risk factors. More medications taken by a patient increases the risk of their health deteriorating [44]. A pharmaceutical column was removed after the previous feature had been extracted. Using the LACE score, we were able to identify patients with high hospitalization risks. As shown in Table 2, the LACE score [42] was designed according to its fully developed phrase using the relevant elements.

Six hundred and seventy-five patients, or nearly 33.87% of all records, had LACE scores indicating that they were at high risk. In total, 63.67% of the study’s participants were in the moderate-risk zone, while 2.46% were in the low-risk zone. LACE alone can be a good predictor of hospital readmission for HF patients and in this study, we have incorporated this LACE feature with other features, which made our model robust.

#### 2.3.5. Feature Scaling

Occasionally, varying ranges and different scaling of data cause the model to run slowly and increase the cost function. Furthermore, all forms of variations and scaling are unlikely to fit the label data effectively [45]. It is also possible that some observations may yield false predictions due to differences in scaling. An ML model should yield a favorable outcome if the quality of the data is guaranteed. The training feature data need to be scaled properly in order to achieve quality data in comparison to the target data. Our study assessed the effectiveness of two normalization methods, min-max normalization, and Z-score normalization to normalize the data [46].

As a result of the min-max normalization method, each feature is scaled with its minimum and maximum value, allowing the data to remain in the 0–1 range. The following formula is used to scale the data (Equation (2)). If X is considered to be a feature value of a certain feature, $X_{scaled}$, $X_{max}$, and $X_{min}$ are scaled output, maximum feature value, and the minimum feature value of that certain feature.

(2) $$X_{scaled}=\frac{X-X_{min}}{X_{max}-X_{min}}$$

Using Z-score normalization, the dataset is transformed into a gaussian distribution. Therefore, it is possible to comprehend the probability of an event occurring within the boundary of a gaussian distribution. This phrase is used in this way (Equation (3)).

(3) $$Z=\frac{X-\mu }{\sigma }$$

Here, **Z**, **X**, μ, and σ are, respectively, the output, feature value, mean, and standard deviation of the variable values.

This approach dramatically improved computation speed by scaling feature values to have a mean of 0 and a standard deviation of 1. Despite this, this strategy may produce outlier values since some observations showed negative instances. In our particular case, min-max scaling performed better than Z-score scaling method while training the model because our dataset contained some outlier values.

#### 2.3.6. Feature Selection

Three different feature selection models were used in this work: XGBoost, Random Forest, and ExtraTrees. Additionally, the feature importance values for each of these selection models have been computed. XGBoost method provides feature importance based on average gain or reduction in the objective function, which provides insights of how a specific feature can contribute in reducing the loss of the model [47]. For Random Forest and ExtraTrees, feature importance is extracted based on mean decrease accuracy, which reduces the impurity in the decision tree when a particular feature is split [48]. In order to achieve the highest accuracy, these feature models were listed in descending order and trained using the logistic regression method’s single feature incrementation, which draws out an optimum number of features needed for the model to score a best result. We can formulate logistic regression as follows:

(4) $$P=\frac{e^{\left(a+bX\right)}}{1+e^{\left(a+bX\right)}}$$

where ***P*** is the logistic regression output, a denotes the expected value of log odds, b stands for change in log odds, and X is the input feature.

**Figure 2:**
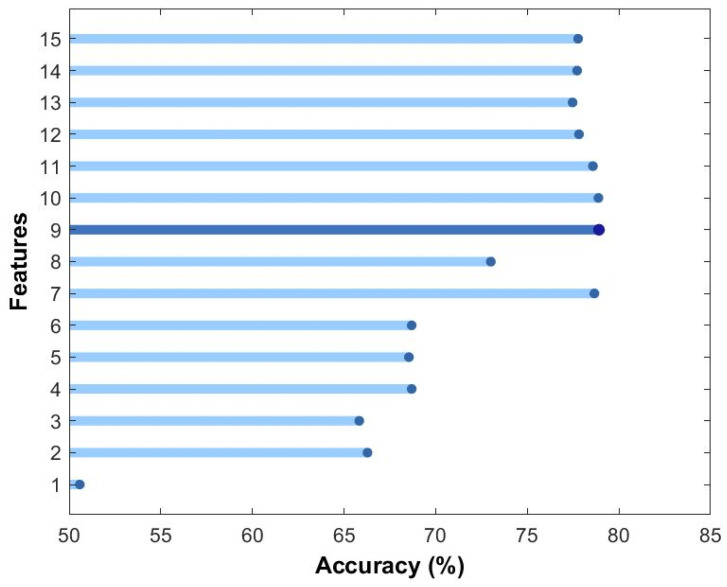
Feature incrementation vs. Accuracy (in %). The number of features is in increments of 1 step each time, and an increase in accuracy of logistic regression classifier is observed. Highest accuracy is observed at 9 features.

In order to draw out the number of features necessary to generate the highest accuracy from the model, the model was trained on the features in increments of 1 feature at time. This method provides valuable insights of how the model performance changes based on the features it is trained on. The outcome of such a method can be observed in Figure 2, which dictates training on 9 features that would present us the best possible performance. Supplementary Table S1 shows the list of features obtained in Figure 2.

**XGBoost**

We conducted a univariate logistic regression analysis to identify the independent variables associated with death. We identified the top 1, top 2, and top 9 features. Gradient-based ensemble tree methods such as XGBoost and GB (Gradient Boosting) are both gradient-descent-based methods that utilize weak learners [47,49,50]. A predictive model was developed by Xu et al. [51] based on XGBoost for ischemic stroke patients readmitted within 90 days of hospital discharge. As a result, they were able to achieve an AUC score of 0.782. XGBoost classifier is trained with a 0.001 learning rate and Logloss as the loss function.

**Random Forest (RF)**

RF is a powerful machine learning algorithm that excels at handling non-linear datasets and outperforms other tree-based algorithms. It utilizes multiple trees with randomized inputs and splits and effectively addresses the problem of overfitting found in decision trees. The RF algorithm solves this issue by averaging the output of each tree and comparing it with the original output. According to Yeshvendra et al. [52], they developed an RF model that achieved 85.81% accuracy in predicting heart disease. This classifier is trained with the following hyperparameters: (bootstrap = True, class_weight = None, criterion = ‘gini’, max_depth = 10, max_features = ‘auto’, max_leaf_nodes = None, min_impurity_decrease = 0.0, min_samples_leaf = 1, min_samples_split = 2, min_weight_fraction_leaf = 0.0, *n*_estimators = 100, oob_score = False).

**ExtraTrees Classifier (ET)**

An ExtraTrees classifier uses the traditional top-down method to create an unpruned decision tree [53]. It involves strong randomization both in attribute selection and in cut-point selection when splitting a node. It differs from other tree-based ensemble methods in that it splits nodes fully randomly and grows trees using the whole training sample instead of bootstrap replicas. The final prediction is made by a majority vote among all three predictions. ExtraTrees reduce variance and bias and are computationally efficient [54]. According to Shafique et al. [55], ExtraTrees were found to be the most accurate models for predicting cardiovascular disease, with an accuracy rate of 96%. ET was trained with the following hyperparameters: (max_depth = 8, min_samples_split =10).

#### 2.3.7. Stacking Machine Learning Model

We have investigated 13 variants of classical ML models for initial prediction performance for hospital readmission of HF patients. Then, we proposed using a stacking ML model that uses 3 classical best-performing ML models. Multiple learners are integrated into stacking-based models, which improve prediction capabilities. This was motivated by a three-layer stacking model demonstrated by Zhang et al. to predict hospital readmission risk [56], where they achieved an AUC of 0.720 outperforming other individual models.

**Figure 3:**
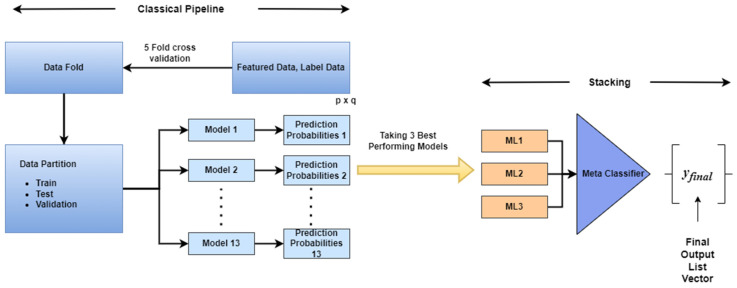
Classical and stacking ML model training framework.

We converted the data using fivefold cross-validation and divided it into training, testing, and validation sets with 80% train and 20% test splits, where 20% of training data used for validation. The training and stacking procedure is graphically shown in Figure 3. The training set had a shape of *p* × q, where *p* represents the number of observations and q represents the top features selected using the top feature approach. A stacking classification model was created based on the predictions of the three best-performing models, allowing for greater precision in categories where the model had previously been unclear. To construct the stacking classification model, we used the best classifier predictions as input to the new meta classifier. A significant improvement in accuracy and performance was achieved with this approach. 

### 2.4. Evaluation Metrics

There were a number of performance evaluation metrics considered for this work, as accuracy alone is not good metric [57,58,59]. As the scores were acknowledged to be the weighted average of all classes, accuracy as the sole metric for evaluation could have skewed the model towards the best-performing class [60]. Furthermore, there are other metrics necessary to explain the AUROC curve in addition to accuracy.

The test set was used to measure the performance metrics of the ML model with the largest area under the receiver operating characteristic curve (AUROC). There were several measures included to compute the performance metrics, including positive likelihood ratios, true positives (TP) and false positives (FP), true negatives (TN) and false negatives (FN), as well as positive predictive value (PPV) [61]. According to AUROC curves, c-statistics are a measure of model discrimination performance for binary classification studies. By comparing patients who actually had the outcome with those who did not, we are able to determine whether a model predicts a higher probability of the outcome. These metrics can be represented mathematically as follows:

(5) $$Accuracy=\frac{TP+TN}{TP+TN+FP+FN}$$

(6) $$Precision=\frac{TP}{TP+FP}$$

(7) $$Recall\;\left(Sensitivity\right)=\frac{TP}{TP+FN}$$

(8) $$F1\;score=\;2\;\;\frac{\mathrm{Precision}\;\mathrm{Recall}}{\mathrm{Precision}+\mathrm{Recall}}$$

(9) $$Specificity\;\left(True\;Negative\;Rate\right)=\frac{TN}{TN+FP}$$

## 3. Results and Discussions

### 3.1. Statistical Analysis

The analysis of this dataset provided significant insights into the characteristics and data that should be focused on when admitting patients at risk. An emergency room admission decision for an HF patient should be made with greater care based on prior diagnostic factors and patient information. It is important to consider the patient’s previous hospitalization data and parameters when prompting them to contact a hospital to prevent exacerbation of the illness. Table 3 summarizes the important feature statistics for the dataset.

In the dataset, there were 900 (45.16%) cases that needed emergency readmission, and 1093 (54.84%) cases were not readmitted. Average discharge day for the subject for readmitted and not readmitted were 10 ± 9.01 (days) and 9 ± 7.06 (days). The cholesterol level for not readmitted cases was 3.77 ± 1.07 (mmol/L) and for the readmitted case 3.66 ± 1.11 (mmol/L). Not readmitted patients had a mean weight of 52 ± 10.7 (kg) and for the other class, the mean was counted as 52.5 ± 11.1 (kg). Additionally, this study indicates the mean reduced Hemoglobin of 4.3 ± 6.22 (%) for the not readmitted class and 4.1 ± 6.0 (%) for the readmitted cases. For both non-readmitted and readmitted cases, both Systolic and Diastolic Blood pressure had a statistically observed level: 133 ± 25.0 (mmHg) and 129 ± 24.2 (mmHg), 77 ± 14.6 (mmHg), and 76 ± 14.2 (mmHg), respectively. Readmitted patients were having 2 ± 3.64 (mg/L) D-dimer in their blood and not readmitted patients were showing 3 ± 6.34 (mg/L), which clearly acted as an indicator by drawing a substantial difference in statistical metrics for both cases. Mitral Valve EMS also proved to be a proper indicator for both the classes, as it showed 6 ± 42.9 (m/s) for not readmitted patients and 3 ± 29.9 for the readmitted patients, although it showed a considerably low correlation value. Readmitted patients were observed to have comparably smaller statistical value range regarding High-Sensitivity Protein having 24 ± 33.5 (mg/L), whereas the patients who were not accounted for emergency readmission showed a value of 26 ± 35.5 (mg/L). We also observed a Glomerular Filtration Rate of 71 ± 36.5 (mL/min/1.73 m^2^) for the patients who did not necessarily need an emergency readmission, where readmitted patients had a filtration rate of 66 ± 36.5 (mL/min/1.73 m^2^). *p*-value < 0.05 was used to check the significance of the features for the classes.

### 3.2. Feature Ranking

**Figure 4:**
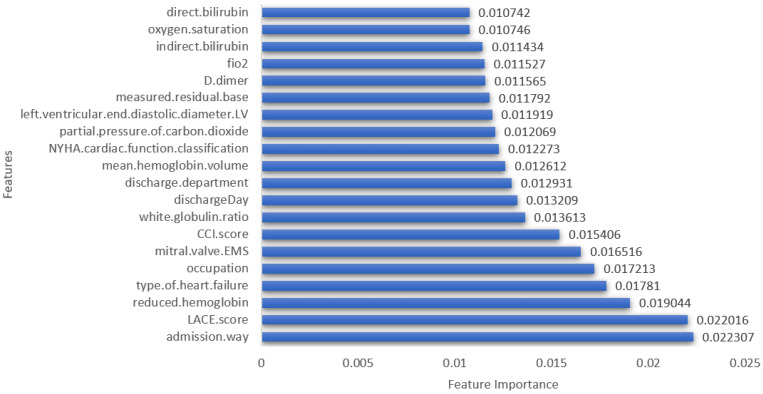
Feature ranking using XGBoost model.

According to the XGBoost ranking algorithm, the top features to identify patients who will require readmission were admission.way, LACE.score, reduced.hemoglobin, type.of.heart.failure, occupation, mitral valve EMS, CCI.score, and white.globulin.ratio. After initial investigation, it was observed that the features identified by XGBoost model outperformed other feature selection-based features. The feature rankings by XGBoost model is shown in ascending order in Figure 4. Therefore, these features were used for the classification investigations. Feature ranking using Random Forest and ExtraTree algorithms are reported in Supplementary Figures S1 and S2.

### 3.3. Classification Performance

*Individual Model Performance:* We implemented 13 variants of classical machine learning models including multilayer perceptron (MLP), Linear Discriminant Analysis (LDA), XGBoost, Random Forest, logistic regression (LR), Support Vector Machine (SVM), ExtraTrees (ET), AdaBoost, K-nearest neighbor (KNN), CatBoost, Gradient Boosting, Light GB (LGB), and ElasticNet. Table 4 summarizes the accuracy, precision, recall, specificity, and F1-score for all the models. Catboost, Adaboost, and Gradient Boosting algorithms are the three top-performing algorithms, which were used to develop the stacking ML model.

*Stacking Model Performance:* The top three performing models were used to create a meta-classifier, and the stacking model was trained based on those results. This last stage of classification provided us with the best results. The stacking ML model provides an overall accuracy, and weighted average of precision, recall, specificity, and F1-score of 89.41%, 90.10%, 89.41%, 87.83%, and 89.28%, respectively. Table 5 summarizes the performance of the stacking ML model, where it is seen that the XGB model as a meta-learner performs the best.

**Figure 5:**
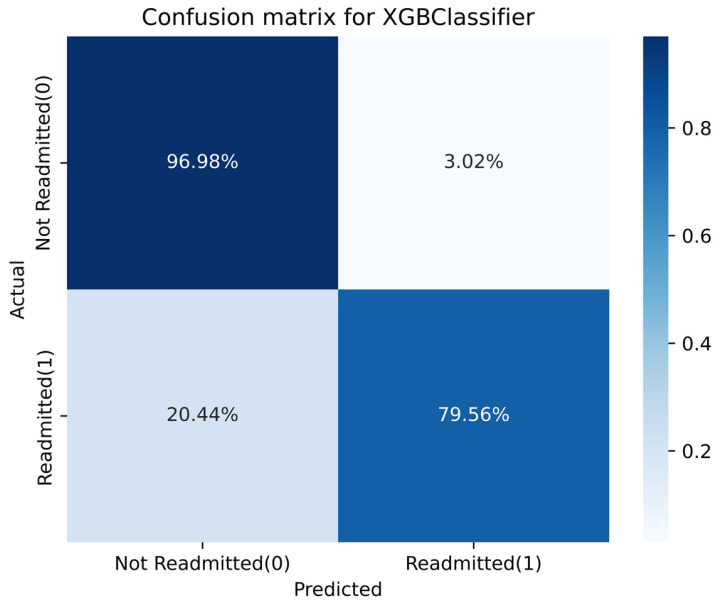
Confusion matrix for the best-performing XGBoost stacking ML classifier.

To assess the quality of our model’s predictive performance, we showed the confusion matrix [62], which summarizes the overall performance of the model based on the performance evaluation metrics introduced earlier. Figure 5 shows the confusion matrix, which shows the correct and incorrect predictions. It can be seen that the model can predict very well the patients who do not need readmission. However, the model suffers for patients who need readmission compared to the other class. The reason of slightly less accurate prediction for admission class might be due to the LACE feature. The LACE scoring method requires the number of emergency department visits in the last 6 months, which is not provided in our dataset. Therefore, we utilized the scoring table to use only the first two scores and leave the rest. There was also a small information gap between the number of days between readmissions and the time to the emergency department in the last six months.

**Figure 6:**
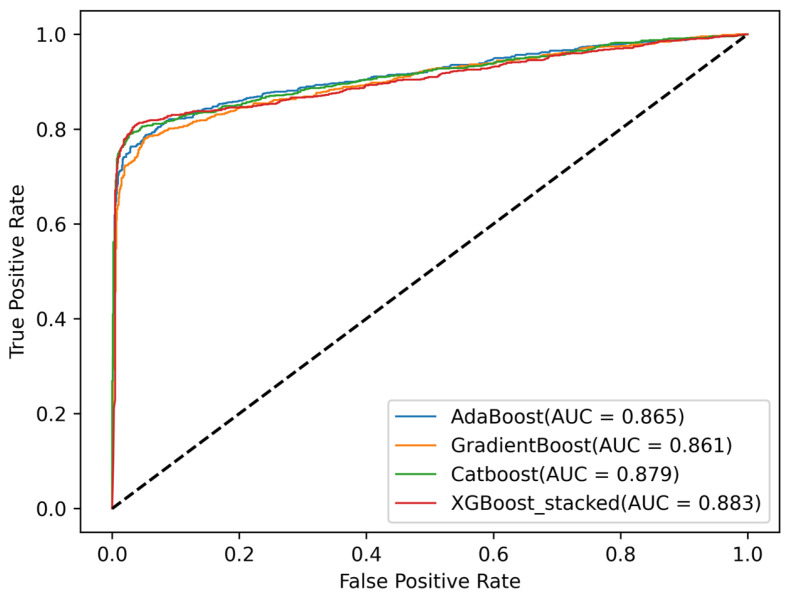
ROC curve for the three best-performing models and the stacked classifier.

The ROC curve represents the degree of separability between binary classes. The larger the area covered by the ROC curve, the more effectively the model utilizes the training dataset. Figure 6 shows the ROC curves for the three best-performing models and the stacking ML model. It can be observed that the AUC scores are very close. XGBoost achieved an excellent AUC score of 0.881, which shows a very good discrimination capability of the model.

### 3.4. Discussion and Comparison with Similar Works

As per the XGBoost ranking algorithm, the foremost attributes for identifying patients who may require readmission comprise admission.way, LACE.score, reduced.hemoglobin, type.of.heart.failure, occupation, mitral valve EMS, CCI.score, and white.globulin.ratio. Upon preliminary examination, it was noted that the features identified by the XGBoost model exhibited greater efficacy than other feature selection-based attributes. Thirteen machine learning models were tested to predict heart failure readmission, and Catboost, Adaboost, and Gradient Boosting were found to be the top-performing models. These three models were used to create a stacking model, which achieved an overall accuracy of 89.41% and weighted average precision, recall, specificity, and F1-score of 90.10%, 89.41%, 87.83%, and 89.28%, respectively. The model performed well in predicting patients who did not require readmission but moderately performed with those who did. XGBoost had the best AUC score of 0.881, indicating excellent discrimination capabilities.

Assessing different models using the same criteria presents a considerable challenge due to the variance in methodologies and variables used to build them. Moreover, the majority of machine-learning-based heart failure readmission prediction models have yet to be verified through an independent prospective cohort of heart failure patients. For our stacking model, it can be seen that the XGB model as a meta-learner performs the best.

The study conducted an in-depth analysis of the experimental results and found that the proposed stacking ensemble model can effectively make use of the predictive abilities of various models to understand the data space and structure. By leveraging this understanding, different models are able to learn from one another, leading to a mutual enhancement in their accuracy of predictions. This suggests that the stacking ensemble model can be beneficial for improving the performance of prediction models, especially when dealing with complex data structures. This claim is further proved by a similar study by Chiu et al. that predicts a model by stacking six classifiers, namely: RF, SVC, KNN, LGBM, Bagging, and Adaboost [63]. According to the stacking model results provided by them, it has achieved an accuracy level of 95.25% alongside an AUROC score of 82.55%. This is consistent with the results of this study. Jing suggested an ML model aimed at precise predictions of one-year, all-cause mortality for a significant number of HF patients. The best prediction performance was achieved by the nonlinear XGBoost model with an AUC score of 0.77 [64]. Chen et al. achieved an accuracy of 0.652 and AUC of 0.634 (95%CI: 0.599–0.646) using LR [65]. Overall, our study highlights the importance of considering the interplay between multiple prediction models in the development of effective machine learning algorithms. It also provides a novel study on predicting emergency readmission of HF patients using only nine biomarkers.

### 3.5. Limitations of Our Work

In terms of dataset, the limitation is that it is obtained from only one center; therefore, the generalizability of the model cannot be guaranteed. The data in the dataset have been merged into a solitary row per hospitalization. Therefore, only superficial portrayals of patient stays are accessible, and time series data for the entire hospitalization are not present. Thus, critical information like medication distribution timings is not provided in the dataset [27]. The study relies on retrospective data where the researchers typically have less control of the dataset variables compared to prospectively collected data.

One of the main limitations of this study is that the features selected by our pipeline did not include any of the important biomarkers of HF: for example, B-type natriuretic peptide (BNP), N-terminal pro-B-type natriuretic peptide (NT-proBNP), Troponin, etc. This requires further investigation and opens up a new area for our future research goal. The reliability of our model is limited to the nine features it currently incorporates, and the absence of any of these features may compromise the accuracy of its predictions. Furthermore, the dataset under scrutiny is geographically confined to a single region, which raises concerns regarding the applicability of our findings to other regions around the world. Given the modest size of our dataset, which comprises approximately two thousand patient records, it is not very suitable to train deep learning models. Consequently, we intend to augment our dataset by aggregating additional datasets pertaining to heart failure, thereby enabling us to develop a comprehensive, one-for-all machine learning pipeline.

## 4. Conclusions

This study aimed to use classical machine learning models based on EHR data to predict emergency readmissions for discharged heart failure patients. Early identification of heart failure patients is crucial to prevent complications and mortality. The proposed method uses the LACE score as a key component to predict subsequent readmissions, enabling healthcare providers to proactively intervene and decrease emergency hospital readmissions while simultaneously improving patient outcomes and reducing healthcare costs. The proposed design accurately predicts emergency readmissions for discharged HF patients with an AUC score of 0.883 using stacking ML model. While the dataset used in this study is limited to one center, our proposed model could still help healthcare providers improve the standard of care for HF patients by fine-tuning the model on a specific population data. Further research is needed to investigate important biomarkers of heart failure not included in the selected features, and augmenting the dataset could enable the development of a more comprehensive framework. The study can be expanded by accumulating multiple datasets from different regions to increase the generalizability of the model. This will also help construct a one-for-all model regardless of race and geographical position. The research demonstrates that using a stacking model can enhance the accuracy of prediction models, particularly when dealing with intricate data structures. Overall, this study is expected to contribute to filling a knowledge gap in multi-feature-based ML models and advance the field of emergency readmission prediction for HF patients, thereby enhancing the quality of heart failure care.

## Figures and Tables

**Table 1:** All types of diseases including HF that are present in the dataset [23].

Disease Name	Yes	No
Myocardial Infarction	1865	143
Congestive Heart Failure	1872	136
Peripheral Vascular Disease	1907	101
Cerebrovascular Disease	1858	150
Dementia	1893	115
Chronic Obstructive Pulmonary Disease (COPD)	1775	233
Connective Tissue Disease	2004	4
Peptic Ulcer Disease	1961	45
Chronic Kidney Disease	1532	474
Hemiplegia	1996	12
Solid Tumor	1969	39
Liver Disease	1923	84

**Table 2:** LACE scoring scheme.

LACE Score Components	Score
**L: Length of stay (days)** **Associated features in the dataset:** **‘dischargeDay’**	
**1**	1
**2**	2
**3**	3
**4–6**	4
**7–14**	5
**≥14**	7
**A: Acuity of admission** **Associated feature in the dataset:** **‘admission.way’**	
**Non-emergency**	0
**Emergency**	3
**C: Charlson comorbidity score** **Associated feature in the dataset: ‘CCI.score’**	
**0**	0
**1**	1
**2**	2
**3**	3
**≥4**	4
**E: Emergency department visits in the preceding 6 months** **Associated feature in the dataset:** **‘return.to.emergency.department.within.6.months’**	
**0**	0
**1**	1
**2**	2
**3** **≥4**	3 4

**Table 3:** Statistics of the different important features for readmitted and not readmitted cases.

Features	Unit	Missing Rate (%)	Not Readmitted (0)	Readmitted (1)	Overall	*p*-Value
			[Mean ± std]	[Mean ± std]	[Mean ± std]	
Neutrophil Ratio	N/A	1	0.756 ± 0.107	0.744 ± 0.10	0.751 ± 0.104	<0.05
Discharge Day	Days	0	9 ± 7.06	10 ± 9.01	9 ± 8.03	0.10
Cholesterol	mmol/L	10	3.77 ± 1.07	3.66 ± 1.11	3.72 ± 1.09	0.06
Sodium	mmol/L	<1	138.5 ± 4.91	137.9 ± 4.87	138.2 ± 4.90	<0.05
Partial Pressure of CO_2_	mmHg	51	36 ± 10.4	35.1 ± 8.13	36 ± 9.56	0.10
Direct Bilirubin	μmol/L	5	9 ± 9.76	9 ± 9.28	9 ± 9.55	<0.05
Albumin	g/L	5	36.3 ± 5.05	36.9 ± 4.87	36.5 ± 4.98	<0.05
Globulin	g/L	5	28.7 ± 6.33	28.4 ± 5.70	28.6 ± 6.06	<0.05
FiO_2_	(%)	0	33.0 ± 5.18	32.33 ± 4.15	32.7 ± 4.76	0.07
Systolic Blood Pressure	mmHg	0	133 ± 25.0	129 ± 24.2	131 ± 24.7	0.07
Cystatin	mg/L	2	1.8 ± 0.971	1.89 ± 0.925	1.8 ± 0.951	0.05
Potassium Ion	mmol/L	51	3.84 ± 0.652	3.98 ± 0.685	3.89 ± 0.669	0.07
White Globulin Ratio	N/A	5	1.32 ± 0.316	1.35 ± 0.30	1.33 ± 0.309	<0.05
D-dimer	mg/L	8	3 ± 6.34	2.0 ± 3.64	2 ± 5.33	0.08
Indirect Bilirubin	μmol/L	5	13 ± 9.57	14.0 ± 8.83	14 ± 9.25	<0.05
Fucosidase	U/L	26	19.3 ± 6.31	19.5 ± 5.78	19.4 ± 6.08	<0.05
Sodium Ion	mmol/L	51	136.4 ± 5.14	135.9 ± 4.71	136.2 ± 4.97	0.09
Left Ventricular End Diastolic Diameter, LV	cm	35	52.4 ± 10.6	54.2 ± 11.3	53.1 ± 10.9	0.12
Eosinophil Ratio	N/A	1	0.017 ± 0.031	0.02 ± 0.031	0.019 ± 0.031	0.05
High Sensitivity Protein	mg/L	53	26 ± 35.5	24 ± 33.5	25 ± 34.7	<0.05
Measured Residual Base	mmol/L	51	−1.8 ± 4.90	−2.1 ± 4.44	−1.9 ± 4.72	<0.05
Glomerular Filtration Rate	mL/min/1.73 m^2^	3	71 ± 36.5	66 ± 36.5	69 ± 36.6	0.05
Mean Hemoglobin Volume	pg	1	29.9 ± 3.28	30.0 ± 3.59	29.9 ± 3.42	<0.05
Mitral Valve EMS	m/s	51	6 ± 42.9	3 ± 29.9	5 ± 38.6	<0.05
Diastolic Blood Pressure	mmHg	0	77 ± 14.6	76 ± 14.2	77 ± 14.5	<0.05
Weight	kg	0	52 ± 10.7	52.5 ± 11.1	52 ± 10.9	<0.05
Basophil Count	×10^9^/L	1	0.030 ± 0.031	0.033 ± 0.028	0.031 ± 0.029	<0.05
Platelet Hematocrit	(%)	5	0.176 ± 0.068	0.17 ± 0.067	0.174 ± 0.068	<0.05
Prothrombin Activity	(%)	2	67 ± 18.3	65 ± 18.6	66 ± 18.4	0.05
Reduced Hemoglobin	(%)	51	4.3 ± 6.22	4.1 ± 6.0	4.2 ± 6.13	0.05
Urea	mmol/L	1	9.4 ± 5.84	9.8 ± 5.16	9.6 ± 5.55	<0.05

**Table 4:** Different evaluation metrics result from 5 folded cross-validated data for different classical machine learning models. Best performances marked in bold.

Model Name	Accuracy	Precision	Recall	Specificity	F1-Score
MLP Classifier	82.19	82.2	82.19	81.27	82.12
Linear Discriminant Analysis	78.02	78.13	78.03	77.94	78.06
XGBClassifier	75.51	75.46	75.51	74.44	75.41
Random Forest Classifier	76.12	76.06	76.11	75.27	76.05
Logistic Regression	78.98	79.01	78.98	78.73	78.99
Support Vector Machine	47.17	58.32	47.16	55.83	34.84
ExtraTree Classifier	70.7	70.66	70.7	69.99	70.67
**AdaBoost Classifier**	**87**	**87.11**	**87.01**	**86.04**	**86.94**
K-nearest neighbor Classifier	70.55	70.47	70.55	69.62	70.48
**Gradient Boosting Classifier**	**84.9**	**84.92**	**84.9**	**84.07**	**84.84**
**CatBoost**	**88.36**	**88.89**	**88.36**	**86.86**	**88.23**
LGB Classifier	80.73	80.78	80.73	79.58	80.62
ElasticNet	78.98	79.02	78.98	78.74	78.99

**Table 5:** Performance of the stacking model with different evaluation metrics.

Model	Accuracy	Precision	Recall	Specificity	F1-Score
MLP Classifier	89.11	90.1	89.11	87.27	88.94
Linear Discriminant Analysis	88.21	88.69	88.21	86.76	88.09
**XGBClassifier**	**89.41**	**90.1**	**89.41**	**87.83**	**89.28**
Random Forest Classifier	89.16	90.07	89.16	87.39	89.01
Logistic Regression	87.36	87.61	87.36	86.16	87.27
Support Vector Machine (SVM)	29.4	29.89	29.41	30.33	29.25
ExtraTrees Classifier	89.06	90.01	89.06	87.25	88.9
AdaBoost Classifier	89.06	89.86	89.06	87.36	88.91
K-nearest Neighbours Classifier	86.4	86.63	86.4	85.2	86.31
Gradient Boosting Classifier	85.85	86.02	85.85	84.72	85.76
CatBoost	89.11	89.92	89.11	87.4	88.96
LGB classifier	88.91	89.74	88.91	87.18	88.75
**ElasticNet**	87.31	87.56	87.31	86.09	87.21

## Data Availability

This work used publicly available datasets collected from Physionet. The dataset can be found here https://doi.org/10.13026/5m60-vs44 [27].

## Supplementary Materials

The following supporting information can be downloaded at: https://www.mdpi.com/article/10.3390/diagnostics13111948/s1, Figure S1: Feature ranking using Random Forest; Figure S2: Feature ranking using Random Forest; Table S1: Ranked 15 features using XGB model.
