# Supplementary material for: Heart Failure Emergency Readmission Prediction Using Stacking Machine Learning Model
Source: Diagnostics (Basel). 2023 Jun 2;13(11):1948. doi: 10.3390/diagnostics13111948 (PMC10252957; doi:10.3390/diagnostics13111948)
Supplement: Supplementary file 1 [file diagnostics-13-01948-s001.zip › diagnostics-2358372-supplementary.pdf]

Supplementary Materials

Supplementary Table S1: Ranked 15 features using XGB model.

Ranking	Name of the Feature
1	Admission way
2	LACE score
3	Reduced Hemoglobin
4	Type of Heart Failure
5	Occupation
6	Mitral Valve EMS
7	CCI score
8	White globulin ratio
9	Discharge Day
10	Discharge Department
11	Mean Hemoglobin Volume
12	NYHA Cardiac Function Classification
13	Partial Pressure of CO2
14	Left Ventricular End Diastolic Diameter LV
15	Measure Residual Base

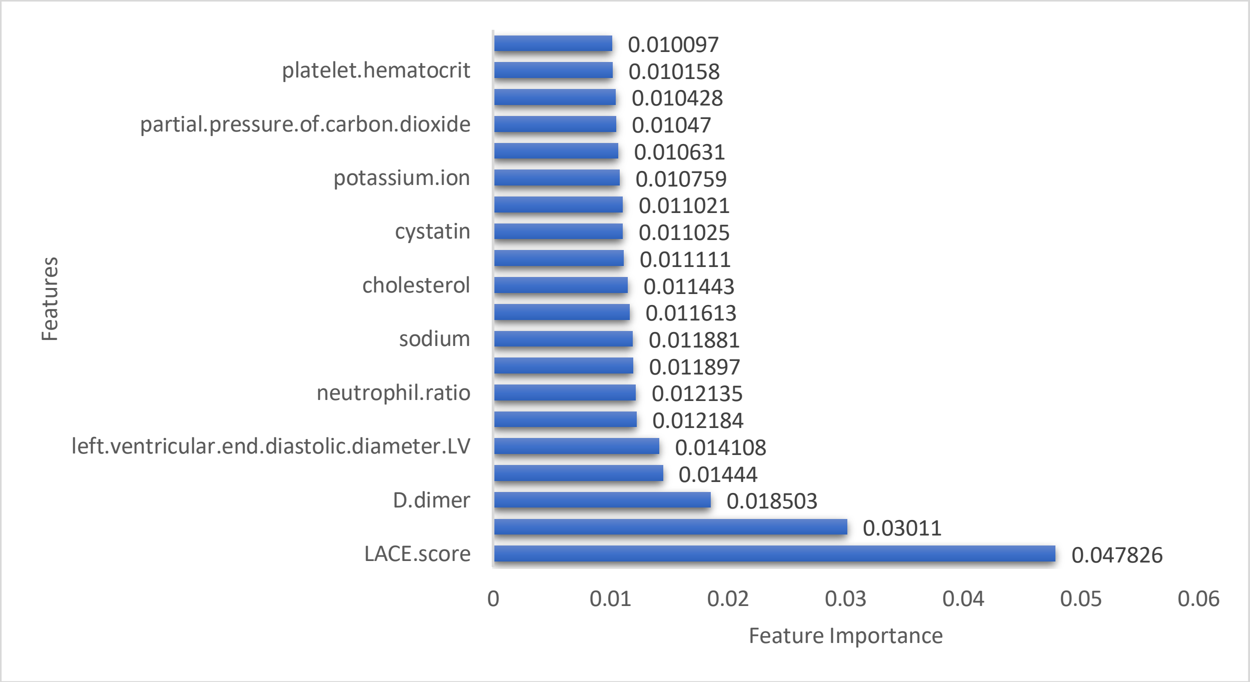

Supplementary Figure S1: Feature ranking using Random Forest.

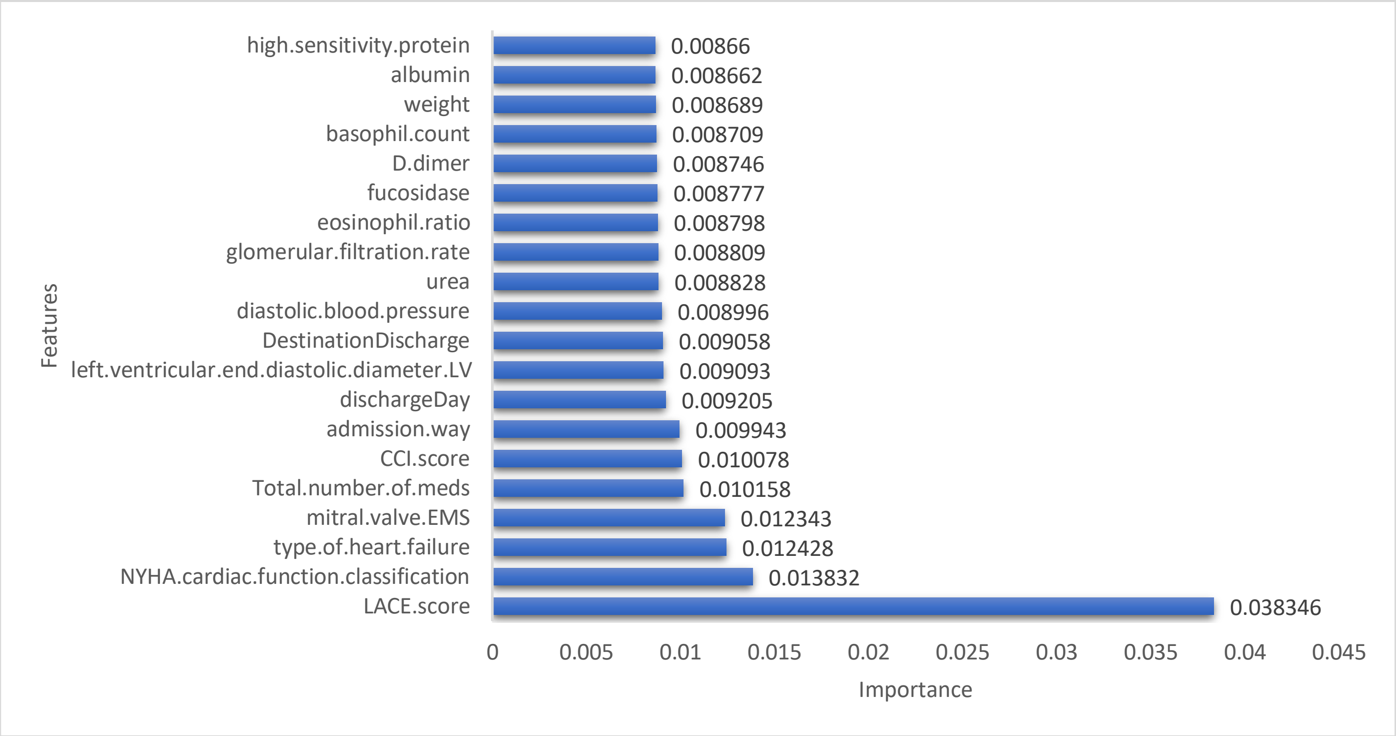

Supplementary Figure S2: Feature Ranking using Extra Tree.
